# Genome-Wide SNP Discovery and Analysis of Genetic Diversity in Farmed Sika Deer (*Cervus nippon*) in Northeast China Using Double-Digest Restriction Site-Associated DNA Sequencing

**DOI:** 10.1534/g3.117.300082

**Published:** 2017-07-27

**Authors:** Hengxing Ba, Boyin Jia, Guiwu Wang, Yifeng Yang, Gilead Kedem, Chunyi Li

**Affiliations:** * State Key Laboratory for Molecular Biology of Special Wild Economic Animals, Institute of Special Wild Economic Animals and Plants, Chinese Academy of Agricultural Sciences, Changchun 130112, China; † GENEWIZ Inc., South Plainfield, New Jersey 07080

**Keywords:** sika deer, ddRAD-seq, SNP discovery, genetic diversity, STACKS, GenPred, Shared Data Resources, Genomic Selection

## Abstract

Sika deer are an economically valuable species owing to their use in traditional Chinese medicine, particularly their velvet antlers. Sika deer in northeast China are mostly farmed in enclosure. Therefore, genetic management of farmed sika deer would benefit from detailed knowledge of their genetic diversity. In this study, we generated over 1.45 billion high-quality paired-end reads (288 Gbp) across 42 unrelated individuals using double-digest restriction site-associated DNA sequencing (ddRAD-seq). A total of 96,188 (29.63%) putative biallelic SNP loci were identified with an average sequencing depth of 23×. Based on the analysis, we found that the majority of the loci had a deficit of heterozygotes (F_IS_ >0) and low values of H_obs_, which could be due to inbreeding and Wahlund effects. We also developed a collection of high-quality SNP probes that will likely be useful in a variety of applications in genotyping for cervid species in the future.

Sika deer are an economically valuable species, with some body parts, such as antler, blood, penis, and placenta, used in traditional Chinese medicine. In order to continue the tradition, people have captured sika deer from the wild on a large scale in Jilin province in the northeastern part of China since the Qing dynasty (c. 1733) (Sheng and Ohtaishi 1993). These animals were gradually introduced into many other regions of China. The original captive population in northeast China is generally considered to be the source of the entire sika deer population on farms throughout China (Sheng and Ohtaishi 1993).

Almost all sika deer raised on farms in northeast China are used for antler (velvet antler) production (Sheng and Ohtaishi 1993; Wu *et al.* 2004). China is currently one of the largest producers of velvet antler in the world and sika deer farming in China is still thriving. Nonetheless, there is still concern about the deterioration of farm-bred sika deer (Wu and Zhang 2001). In recent decades, people have been trying to improve velvet antler yield through long-term improved management on farms (McCullough *et al.* 2009). Whether a healthy genetic diversity can be maintained is an open question. Therefore, any genetic management requires detailed knowledge of the genetic diversity.

The genetic diversity of the sika deer wild population has been investigated using two major sources of genomic variation. Mitochondrial genes, such as cytochrome b and the control region, have proven highly informative for investigations into the classification and phylogeny of sika deer with maternal haplogroups (Wu *et al.* 2004, 2005; Lu *et al.* 2006; Takiguchi *et al.* 2012; Krojerova-Prokesova *et al.* 2013; Ba *et al.* 2015, 2016). Autosomal microsatellites have been used extensively to estimate levels of genetic diversity (Tamate *et al.* 2000; Thevenon *et al.* 2004; Krojerova-Prokesova *et al.* 2013; He *et al.* 2014). Although highly polymorphic markers can be very informative, in particular when assessing recent demographic bottlenecks (endangered and captive-bred species), the previous data are not sufficient to provide a good understanding of the level of genetic diversity in the farmed population in China. This is the first study to report levels of genetic diversity within sika deer using single nucleotide polymorphism (SNP) markers on a global scale.

Their genomic abundance and amenability to cost-effective, high-throughput genotyping, has meant that SNPs are now the most widely used class of genetic markers. Use of genome-wide SNPs constitutes an important genetic tool for investigating genetic diversity in livestock animals (Groeneveld *et al.* 2010). Although genome-wide SNP discovery in white-tailed deer (Seabury *et al.* 2011) and hog deer (Wang *et al.* 2017) has been reported, development of this tool in deer has been hampered in part by the substantial duplicate regions in large parts of the deer genome, and by the lack of a reference genome sequence. Fortunately, double-digest restriction site-associated DNA sequencing (ddRAD-seq) technology (Peterson *et al.* 2012) can provide a flexible and inexpensive platform for the simultaneous discovery of tens of thousands of SNP markers in model and nonmodel organisms. While several SNP genotyping tools have been developed for SNP discovery, including GATK (DePristo *et al.* 2011), STACKS (Catchen *et al.* 2011, 2013), SAMtools (Li 2011) and RADtools (Baxter *et al.* 2011), STACKS is recommended for ddRAD-seq projects, as it uses a multinomial-based likelihood model to call SNPs, which incorporates a bounded SNP model.

In this study, we employed the ddRAD-seq technology to achieve the first genome-wide SNP discovery for farmed sika deer that are widely distributed in northeast China. The STACKS pipeline was applied for SNP calling. Using newly developed markers, we investigated the genetic diversity of farmed populations. We also developed a collection of SNP probes for genotyping in the future.

## Materials and Methods

### Sample collection

A total of 42 unrelated animals were selected from eight farms (farm codes: AD, CB, DF, NM, SP, SY, XF, and XK) in northeast China, with six from NM and XK farms and five from the other six farms. Whole-blood samples were extracted from the jugular vein using EDTA vacuum tubes and were stored at −20 °C until DNA extraction. The genomic DNA extractions from whole blood were performed using a blood DNA kit (Qiagen) according to the manufacturer’s instructions. Each DNA sample was evaluated by gel electrophoresis for the presence of high-molecular-weight DNA and then stored at −80°C until ddRAD-seq library construction.

### ddRAD-seq library preparation and sequencing

The procedure was performed as described in previous studies (DaCosta and Sorenson 2014) with some modifications. First, the double-digest reactions were carried out in a volume of 25 μl containing ∼0.8 μg of genomic DNA, 5U of PstI and MseI, and 1× buffer 3.1 (NEB). The reaction mixture was incubated at 37 °C for 2 hr and 65 °C for 30 min Amplification and sequencing adapters with a unique barcode (5 or 6 bp) were ligated on to the digested DNA. Each sample was then amplified via PCR in a 50 μl reaction volume, containing 70–100 ng of adaptor-ligated DNA fragments, and amplified with 22 cycles following the manufacturer’s protocol. Samples were run on a 2% agarose gel, and DNA in the 300–450 bp size range (with indices and adaptors) was excised using a gel extraction kit (Qiagen). Each sample library was pooled in equal amounts and quantified using Agilent 2100 (Agilent Technologies) and real-time quantitative PCR, and then paired-end 101 bp sequencing was performed using the Illumina HiSeq4000 platform (BGI, Shenzhen, China).

### Available sika deer genome reference

The available sika deer genome reference (SK-REF) included 387,939 contigs, comprising a total of 2.73 Gbp. All sequences were longer than 500 bp, with a N50 length of 38.4 kbp. The percentage of repeat sequences detected by Repeatmasker-4.0.6 (http://www.repeatmasker.org) was 44.14% and the GC level was 42.11% (H. Ba, unpublished data).

### RAD-seq data analysis and SNP identification

Given the main quality score parameters (-c -q -r) of the process_radtags program in STACKS v.1.41 (Catchen *et al.* 2011, 2013), raw reads were filtered and separated by barcode. The filtered paired-end reads were uniquely mapped to SK-REF using BOWTIE v2.0 (Langmead and Salzberg 2012) (disallowing gaps and suppressing unpaired and discordant alignments), followed by SAMtools v1.2 (Li 2011) to convert to a BAM file. Putative orthologous loci were assembled for each animal using the BAM file as the input of the pstacks function with the minimum depth of coverage above 5. Two mismatches were allowed between animals when assembling the catalog of ddRAD loci using the cstacks program. Matches of individual ddRAD loci to the catalog were searched using the sstacks program. Loci that have low depth of coverage or high sequencing error will exhibit poor log likelihood scores that are highly negative. The rxstacks program (-conf_filter -conf_lim 0.75 -prune_haplo -lnl_lim -8.0) was used to make corrections to genotype calls in individuals based on data from a population-wide examination. Subsequently, the cstacks and sstacks programs were again run to rebuild and match to the catalog. The populations program was used to filter the dataset to contain SNPs found in at least 75% of individuals and with a minor allele frequency ≥0.05.

### Estimates of genetic diversity within population

Observed heterozygosity (H_obs_), expected heterozygosity (H_exp_) and inbreeding coefficient of an individual relative to the subpopulation (F_IS_) were calculated using the populations program in STACKS v.1.41. Deviation from the Hardy–Weinberg equilibrium (HWE) was assessed by performing a Fisher’s exact test with the HardyWeinberg R package (Graffelman and Camarena 2008) for each SNP marker. The False discovery rate correction (Benjamini–Hochberg) was performed using the p.adjust program in the R package.

### Population structure analysis

Bayesian clustering analysis implemented in STRUCTURE 2.3.4 (Pritchard *et al.* 2000) was used for estimating the number of populations/groups (*k*) represented by the dataset. Three iterations were run per *K* (number of populations) for *K* = 2 or *K* = 3 using an admixture model. Each run consisted of a burn-in of 100,000 MCMC steps, followed by 500,000 replications. Population structure was also examined by carrying out principal component analysis using SMARTPCA within EIGENSOFT (Patterson *et al.* 2006).

### Development of SNP probes for genotyping assay

A custom script was run to develop high-quality SNP probes. In summary, sufficient 50 bp flanking sequences on either side of the SNPs were derived from the SK-REF genome and then were filtered according to four filtration criteria: (a) no repetitive sequences; (b) SNP-free within flanking sequence; (c) one of two alleles in accordance with the base in the reference; (d) flanking sequence on the SK-REF ≥50bp.

### Data availability

Raw (adapter trimmed) Illumina ddRAD-seq sequences: NCBI Short Read Archive (project accession: SRP105008). Statistics describing different properties of each sequenced individual and all high-quality SNP probes are available in the Supplemental Material, Table S1 and Table S2.

## Results

### ddRAD sequencing

After trimming the barcodes and filtering low-quality bases, ddRAD sequencing generated a total of over 1.45 billion high-quality paired-end reads (288 Gbp) across the 42 animals. Of these clean reads, >0.93 billion (65.63%) were aligned uniquely to the SK-REF genome. As variation among animals is often observed in a pooled ddRAD sequencing library, the number of aligned reads per individual ranged from 0.55 to 5.3 million (1.01–9.78 Gbp, with an average of 4.1 Gbp) (Table S1).

### SNP discovery

Using the Stacks pipeline, we initially obtained 7,576,300 candidates of the ddRAD loci from all individuals. Quality filtering (see Methods) reduced this to a total of 324,564 (4.28%), which were shared by >75% of the individuals, corresponding to genomic size of 0.032 Gbp (∼1.1% of the genome) (Table 1). Within these 324,564 ddRAD loci of ∼100 bp, we detected 96,188 (29.63%) putative biallelic SNPs with an average sequencing depth of 23×. For each individual, the number of SNPs varied from 56,388 (58.6%) to 89,845 (93.4%), and the sequencing depth ranged from 9× to 50×. The number of identified heterozygous SNPs varied from 11,780 to 22,641 with an average of 19,078, corresponding to 0.38–0.71 heterozygous SNPs per kilobase pair with an average of 0.61 detected per individual animal (Table S1). With a read rate within individuals averaging 83%, this suggests that the rate could be ∼0.74 per kbp.

**Table 1:** Filtering results for STACKS pipeline

Quality Filtering Steps	Number of ddRAD Loci	%
Populating observed ddRAD loci for 42 animals	7,576,300	100.0
Removing ddRAD loci below the minimum depth of 5×	7,456,091	98.4
Removing ddRAD loci below the log likelihood threshold of −8	1,989,000	26.3
Removing ddRAD loci below the minimum constraint of 32 (75%) individuals	324,564	4.3
Putative biallelic SNP (MAF ≥0.05)	98,166	1.3

**Figure 1:**
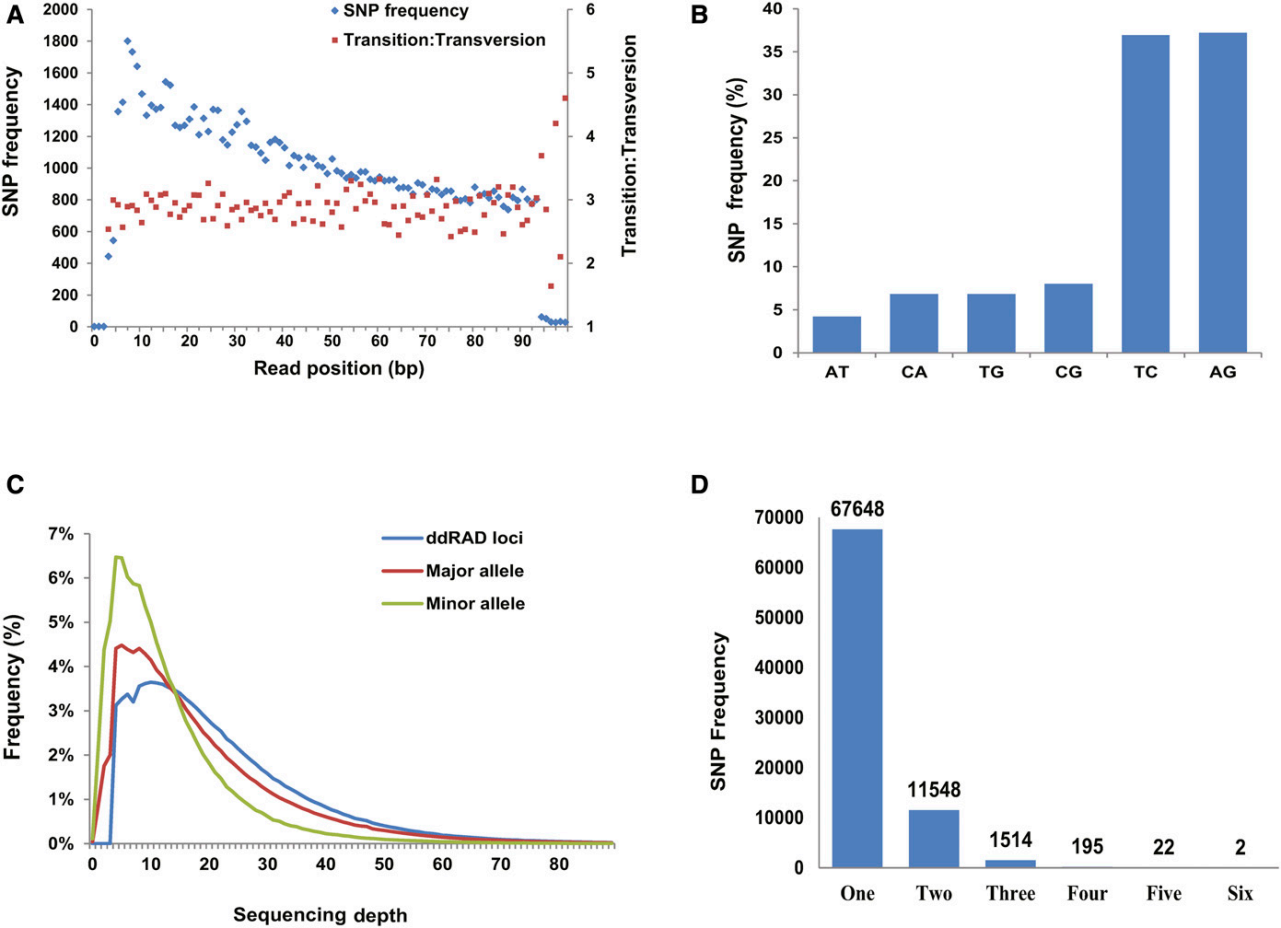
Evaluation of putative SNP quality. (A) Distribution of SNP frequency on ddRAD tag positions. Distribution of the identified Ts/Tv is also illustrated. (B) Distribution of six types of substitutions. (C) Distribution of SNP frequency in major allele, minor allele, and ddRAD loci against their sequencing depth. (D) Distribution of SNP frequency of the number of SNPs in ddRAD loci tags.

Through evaluation of SNP frequency, substitution types and sequencing depth, the results showed that: (a) SNP frequency decreased from 5′ to 3′ end in all loci excluding both ends; however, transition/transversion (Ts/Tv) ratios were very stably distributed over the loci except for the last five positions at the 3′ end (Figure 1A); (b) frequency of A/T substitution is relatively less than other types of substitutions (Figure 1B), which could be attributed to lower coverage in AT-rich region; (c) minor alleles had relatively lower sequencing depth, but the difference between the depths of the minor and major alleles was not statistically significant (Wilcox paired test *P* value = 0.056) (Figure 1C); (d) a majority of loci contained one SNP and only 1733 (2.14%) were identified with >2 SNPs (Figure 1D).

### Genetic diversity

**Figure 2:**
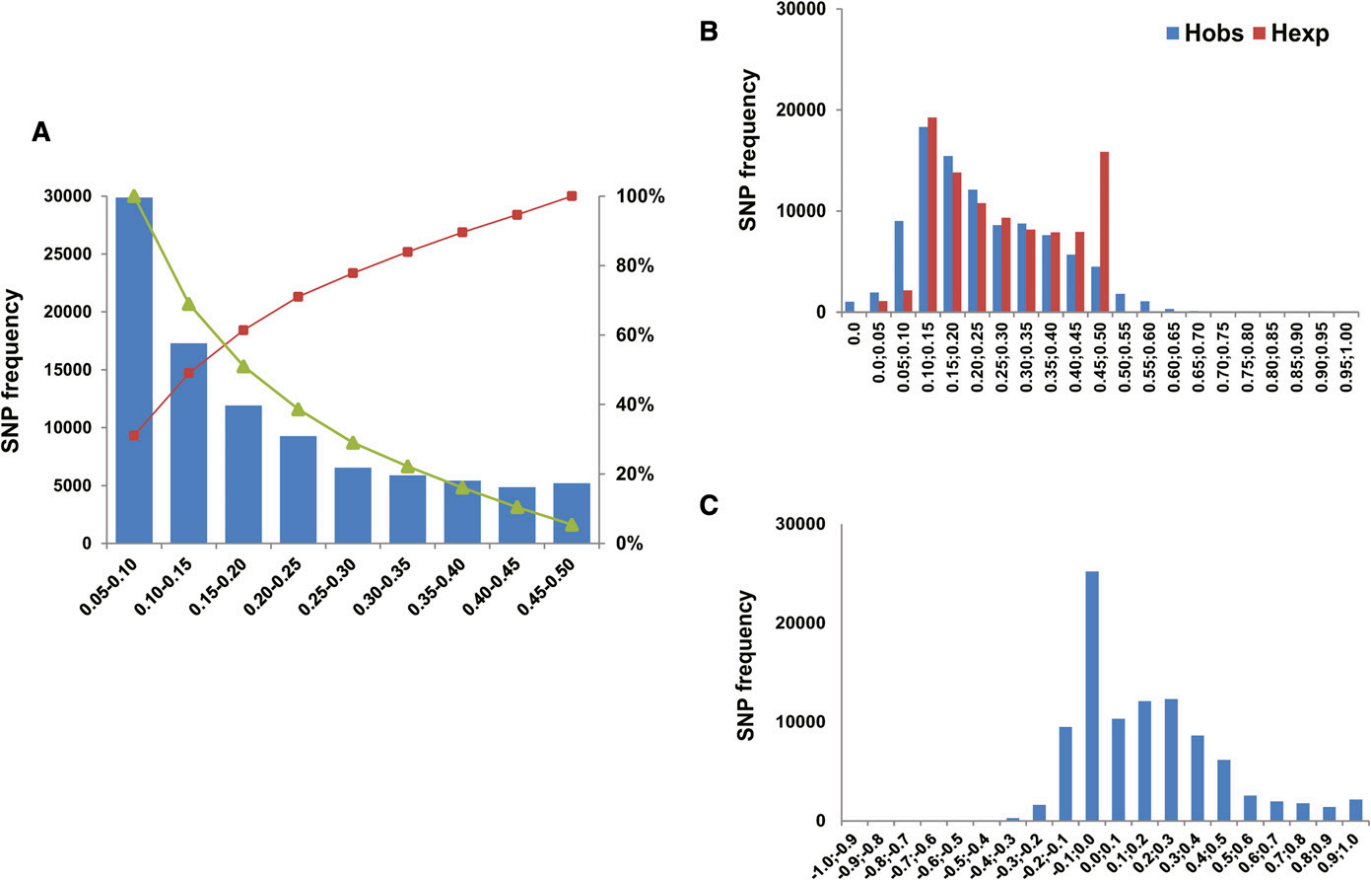
Population genetic parameters for putative SNP loci. (A) Frequency of SNPs across the MAF bins. The line depicts the cumulative density function across the MAF bins. (B) Frequency of SNPs across the heterozygosity bins. (C) Frequency of SNPs across the F_IS_ bins.

Genetic parameters evaluated using putative SNPs included MAF, H_obs_, H_exp_, F_IS_, and deviation from HWE for the studied population. The distribution of MAF showed that the SNPs with MAF <0.15 were overrepresented at nearly half of the SNPs (49.0%), and SNPs were evenly distributed in high-MAF bins ranging from 0.25 to 0.5 (29%) (Figure 2A). The average MAF of all SNP loci was 0.20.

Of 96,188 SNPs, 11,876 (12.35%, at *P* ≤0.05) were shown to deviate from HWE. Following application of the Benjamini–Hochberg correction, 8205 (5.53%) SNPs remained significant (*P* ≤0.05). The excess of H_obs_ values was observed in the frequency distribution from 0 to 0.15, and the deficit was from 0.4 to 0.5 (Figure 2B). The deficit of heterozygotes (F_IS_ >0) is shown in Figure 2C, indicating a high level of inbreeding. The average H_obs_, H_exp_, and F_IS_ calculated across all 96,188 SNPs were 0.24, 0.28, and 0.16, respectively.

#### Genetic structure:

**Figure 3:**
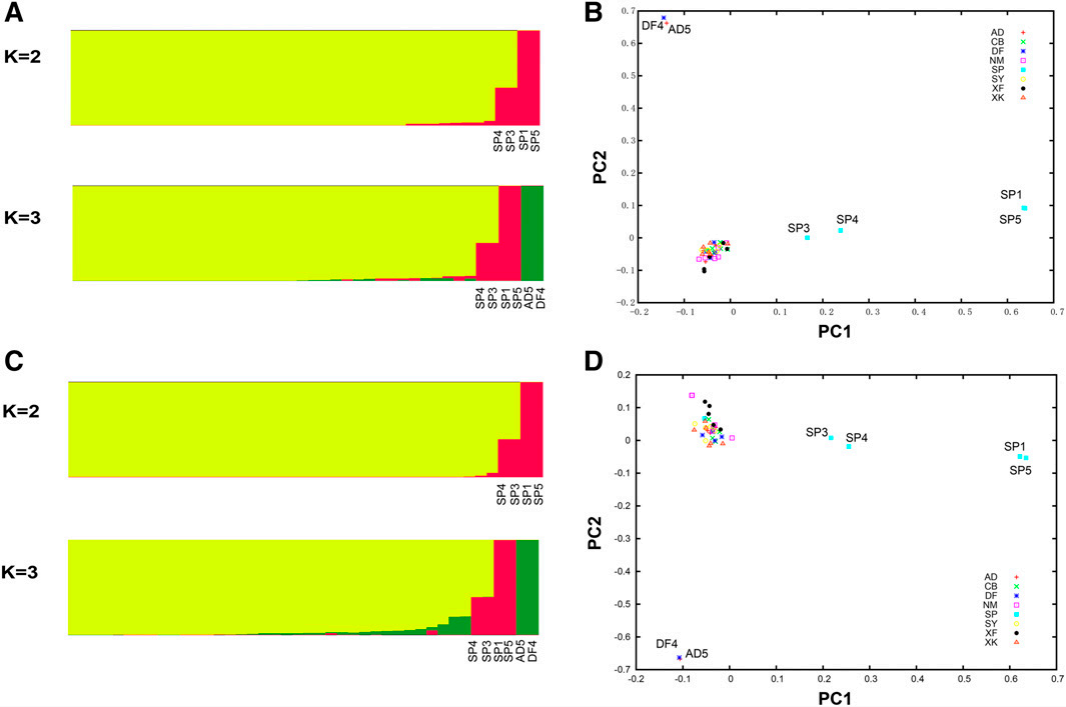
Population genetic structure. (A) STRUCTURE analysis for *K* = 2 and 3 for 96,188 SNPs. (B) PCA analysis for 96,188 SNPs. (C) STRUCTURE analysis for *K* = 2 and 3 for 13,739 SNPs. (D) PCA analysis for 13,739 SNPs.

In order to assess whether there is a stratification within the farmed sika deer population, population structure was estimated using all SNPs (96,188) across >32 animals (≥75%). Since the STRUCTURE model assumes that the loci are independent and at HWE, we also performed STRUCTURE analysis on a filtered SNP dataset (13,739) at HWE (*P* ≥0.05) with one SNP on each ddRAD loci across >38 animals (≥90%). Graphic displays of the consistent results from two SNP datasets provided a meaningful explanation of the genetic structure and levels of admixture for the farmed populations (Figure 3, A and C). At *K* = 2, the two clusters were distinct; four animals (SP1, SP3, SP4, and SP5) were clearly distinguished and, of these, SP3 and SP4 represented an admixture pattern (two clusters of genetic background). At *K* = 3, another cluster of two animals (AD5 and DF4) was evident. Similar results were also observed using the PCA approach based on these two SNP datasets. Although PC1 and PC2 accounted for only 2.4% and 1.9% of total variation, respectively, four animals (SP1, SP3, SP4, and SP5) were clearly distinguishable based on PC1, and two animals (AD5 and DF4) based on PC2 (Figure 3, B and D).

### SNP probes

**Figure 4:**
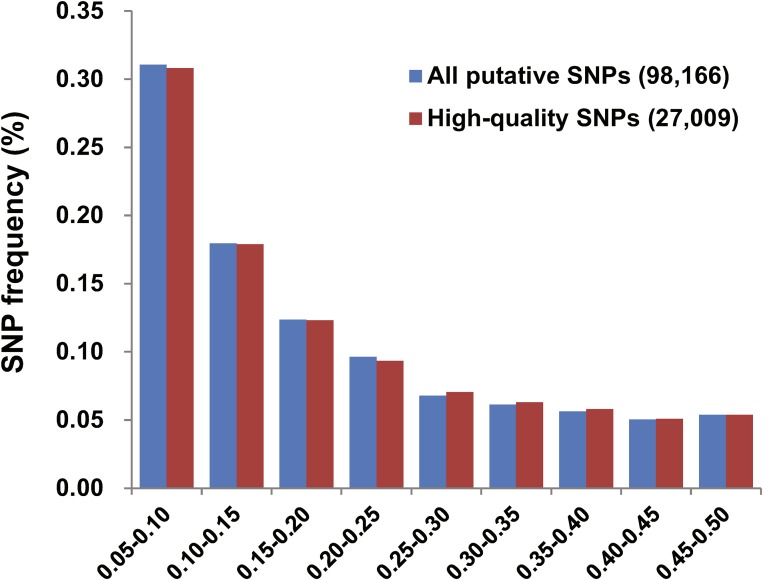
Comparison of SNP frequency distribution between all putative SNPs and high-quality SNPs.

We selected 27,009 high-quality SNPs probes according to four filtration criteria. The Ts/Tv ratio was 3.72. The MAF frequency distribution was accordant between the 96,188 and 27,009 SNP datasets (Figure 4), suggesting that the repetitive sequences have no effect on the change of MAF frequency distribution for the discovered SNPs. These SNP probes (101 bp) were matched with the bovine genome assembly (UMD3.1) via BLASTN, and their alignment resulted in 58.42% (15,778/27,009) probes producing 50,447 *E*-value informative hits (*E* value 1e^−20^). As expected, 96.24% (15,185/15,778) of these matched SNP probes produced one unique alignment to a bovine genome, with an average identity of 94.39%. Only 3.76% (593/15,778) probes hit multiple cattle genome positions (35,262).

## Discussion

The ddRAD-seq technique is an efficient and cost-effective means of SNP discovery, providing thousands of high-quality SNPs, even in the absence of an available genome sequence (Peterson *et al.* 2012). Using the ddRAD-seq technique, we reported here the first genome-wide set of 96,188 novel SNPs in sika deer from 42 unrelated animals selected from eight farms.

In order to evaluate the application of SNPs reported in our study, the SNP quality was screened based on the data characteristics of next-generation sequencing technology. Sequencing errors are often found at the 3′ end of the sequence reads (Dohm *et al.* 2008). If a substantial proportion of the SNPs in the dataset were the result of sequencing errors, the number of SNPs toward the end of the reads should increase. However, our results showed that the number of SNPs identified actually decreased from the 5′ to the 3′ end (Figure 1A), indicating that base errors at the 3′ end were not identified as SNPs. An underrepresentation at both ends of the reads (positions 1–3 and 95–100) was attributed to the *Mse*I and *Pst*I restriction sites and the effect of barcode trimming. However, the Ts/Tv ratio is very stably distributed over the loci except at the very ends of the reads, and was not affected by the relatively higher base error rate at the ends of the reads.

The expected Ts/Tv ratio can vary with the targeted region (*i.e.*, whole genome, exon, specific genes) and can also vary greatly in the CpG islands and GC content of the region (Dohm *et al.* 2008). For example, the Ts/Tv ratio of 3.5 is typical of coding regions in human genomes (Le and Durbin 2011). Higher Ts/Tv ratios are also observed in other reduced representation libraries for SNP discovery (Kraus *et al.* 2011; Le and Durbin 2011). Therefore, it is not unexpected that the observed Ts/Tv ratio is relatively higher in the SNP dataset. The number of A/T substitutions is relatively lower than other types of substitutions (Figure 1B). This could be attributed to the fact that higher coverage needs to be attained to discover real SNPs in AT-rich regions (Dohm *et al.* 2008). Matching the base content of a restriction site to a reference genome can also influence substitution types (Davey *et al.* 2011).

To date, the SK-REF database comprises ∼90% of the sika deer genome, and is incomplete. The unique alignment of ddRAD reads does not guarantee that there is no other similar sequence in the remainder of the genome. This type of genome mapping could result in abnormally high coverage in the paralogous or duplicated sequence, which will likely generate a large number of false-positive SNPs with excessive heterozygotes (Malhis and Jones 2010). Our results show that the depths of almost all alleles fall into a reasonable range (less than threefold average depth, 42× for minor alleles and 60× for major alleles) (Figure 1C). This is an acceptable depth range for SNP discovery that had been identified in other reduced representation libraries of sequencing projects, including cattle (Eck *et al.* 2009), porcine (Amaral *et al.* 2009) and turkey (Kerstens *et al.* 2009). We did not observe excess heterozygotes, in which the number of SNPs with H_obs_ >0.5 is relatively lower in the SNP dataset (Figure 2B). In addition, only 1733 (2.14%) ddRAD loci were identified with >2 SNPs, which was also a good indicator of correct homologous loci matching (Figure 1D).

The average frequency of heterozygous SNPs per kilobase pair is 0.74 in the genome of sika deer individuals. As a comparison, this value is slightly higher than that of milu (a highly endangered species that is susceptible to genetic drift and inbreeding because of small population size), but almost <2 times those of cow and panda (Table 2). The relatively low level of genetic variability in the farmed population could be due to ongoing inbreeding within a narrow gene pool and genetic drift. Additionally, the 42 individuals sequenced were from eight farmed populations that may be small and divergent in allele frequencies. For instance, almost 5.53% (8205) of SNPs (Benjamini–Hochberg correction *P* ≤0.05) deviate from HWE, which may be caused by the different subpopulations sampled. The higher values of F_IS_ and the lower-than-expected values of H_obs_ could be also explained by subpopulation structure (Wahlund effect). The STUCTURE and PCA results further provide evidence of subpopulation structure in farmed sika deer, despite the lack of overall variation and the fact that the PC1 and PC2 accounted for such a small proportion (2.4% and 1.9%, respectively) of the total variation. The small-scale farming population and the application of the technique of artificial insemination over a long period have encouraged inbreeding and genetic drift for velvet antler production. Such population events also compromise the genetic structure of the original population. Hence, our results could help in investigating the structure of farmed populations so such disadvantages can be better managed.

**Table 2:** Information of SNP frequency among four species

Species	Study Population	Sample Size	Average SNP Frequency in Individual (kb^−1^)	Average Depth	Reference
Sika Deer	Deer from eight farms	42	0.74	23×	This study
Milu (Pere David’s Deer)	Captive population	5	0.51	NA*^a^*	Zhu *et al.* (2016)
Panda	Wild population	34	1.32	4.7×	Zhu *et al.* (2016)
Holstein-Friesian Cows	Breeding population	32	1.35	14.3×	Szyda *et al.* (2015)

a NA, not applicable.

SNP markers are not only readily applied to population genetic analyses, but also suitable for parentage testing and assessment of breed composition. Genome-wide association analysis may offer the opportunity to identify the genomic regions and mutations that underpin production traits such as velvet antler weight. One attractive feature of association studies is that pedigrees are not necessary, so potentially a larger number of the farmed animals may be amenable to this type of analysis (Seabury *et al.* 2011). However, high-quality phenotypes are critical, and this will require major investment.

There may be potential to apply genomic selection for the genetic improvement of farmed sika deer. The small effective population size may make this an attractive proposition. The option of introducing a different subpopulation would also enable the exploitation of heterosis (hybrid vigor).

Overall, we have discovered ∼27,009 high-quality SNPs in 1% of the sika genome using ddRAD-seq technology. A total of 58% (15,778/27,009) probes were uniquely matched on to the bovine genome (BLASTN E value 1e^−20^), which could be an indication of the sharing of SNP genotypes between the genomes. Only 593 SNP probes of sika deer could be aligned multiple times to the bovine genome, which may represent a duplicated and/or expanded region on the cattle genome. In comparison, Haynes and Latch (2012) successfully genotyped a lower proportion of loci (39%) in mule deer using BovineSNP50 BeadChip, and Powell *et al.* (2016) directly captured 60% exon data from bovine genome on mule deer genome, matching rates almost concordant with our data. As deer and cattle diverged 27–32 MYA (Hassanin and Douzery 2003; Guha *et al.* 2007), the matching of sika deer SNPs discovered in the bovine genome would likely be useful to study genomic evolution and phylogenetic analysis across species within Cervidae, given the high cross-species matching success rate. Although, to date, there is no valuable reference genome within the Cervidae family and map positions are unknown, SNP assays derived from nonrepetitive loci (contigs) could be designed for high-density genotyping across cervid species, providing a reasonable genome analysis tool for future research.

## Supplementary Material

Supplemental material is available online at www.g3journal.org/lookup/suppl/doi:10.1534/g3.117.300082/-/DC1.

## References

[bib1] Amaral A. J. Megens H. J. Kerstens H. H. Heuven H. C. Dibbits B., 2009 Application of massive parallel sequencing to whole genome SNP discovery in the porcine genome. BMC Genomics 10: 374. 19674453 10.1186/1471-2164-10-374 PMC2739861

[bib2] Ba H. Yang F. Xing X. Li C., 2015 Classification and phylogeny of sika deer (Cervus nippon) subspecies based on the mitochondrial control region DNA sequence using an extended sample set. Mitochondrial DNA 26: 373–379. 24063645 10.3109/19401736.2013.836509

[bib3] Ba H. Wu L. Liu Z. Li C., 2016 An examination of the origin and evolution of additional tandem repeats in the mitochondrial DNA control region of Japanese sika deer (Cervus Nippon). Mitochondrial DNA A DNA Mapp. Seq. Anal. 27: 276–281. 24621225 10.3109/19401736.2014.892077

[bib4] Baxter S. W. Davey J. W. Johnston J. S. Shelton A. M. Heckel D. G., 2011 Linkage mapping and comparative genomics using next-generation RAD sequencing of a non-model organism. PLoS One 6: e19315. 21541297 10.1371/journal.pone.0019315 PMC3082572

[bib5] Catchen J. Hohenlohe P. A. Bassham S. Amores A. Cresko W. A., 2013 Stacks: an analysis tool set for population genomics. Mol. Ecol. 22: 3124–3140. 23701397 10.1111/mec.12354 PMC3936987

[bib6] Catchen J. M. Amores A. Hohenlohe P. Cresko W. Postlethwait J. H., 2011 Stacks: building and genotyping Loci de novo from short-read sequences. G3 1: 171–182. 22384329 10.1534/g3.111.000240 PMC3276136

[bib7] DaCosta J. M. Sorenson M. D., 2014 Amplification biases and consistent recovery of loci in a double-digest RAD-seq protocol. PLoS One 9: e106713. 25188270 10.1371/journal.pone.0106713 PMC4154734

[bib8] Davey J. W. Hohenlohe P. A. Etter P. D. Boone J. Q. Catchen J. M., 2011 Genome-wide genetic marker discovery and genotyping using next-generation sequencing. Nat. Rev. Genet. 12: 499–510. 21681211 10.1038/nrg3012

[bib9] DePristo M. A. Banks E. Poplin R. Garimella K. V. Maguire J. R., 2011 A framework for variation discovery and genotyping using next-generation DNA sequencing data. Nat. Genet. 43: 491–498. 21478889 10.1038/ng.806 PMC3083463

[bib10] Dohm J. C. Lottaz C. Borodina T. Himmelbauer H., 2008 Substantial biases in ultra-short read data sets from high-throughput DNA sequencing. Nucleic Acids Res. 36: e105. 18660515 10.1093/nar/gkn425 PMC2532726

[bib11] Eck S. H. Benet-Pages A. Flisikowski K. Meitinger T. Fries R., 2009 Whole genome sequencing of a single Bos taurus animal for single nucleotide polymorphism discovery. Genome Biol. 10: R82. 19660108 10.1186/gb-2009-10-8-r82 PMC2745763

[bib12] Graffelman J. Camarena J. M., 2008 Graphical tests for Hardy-Weinberg equilibrium based on the ternary plot. Hum. Hered. 65: 77–84. 17898538 10.1159/000108939

[bib13] Groeneveld L. F. Lenstra J. A. Eding H. Toro M. A. Scherf B., 2010 Genetic diversity in farm animals–a review. Anim. Genet. 41(Suppl. 1): 6–31. 20500753 10.1111/j.1365-2052.2010.02038.x

[bib14] Guha S. Goyal S. P. Kashyap V. K., 2007 Molecular phylogeny of musk deer: a genomic view with mitochondrial 16S rRNA and cytochrome b gene. Mol. Phylogenet. Evol. 42: 585–597. 17158073 10.1016/j.ympev.2006.06.020

[bib15] Hassanin A. Douzery E. J., 2003 Molecular and morphological phylogenies of ruminantia and the alternative position of the moschidae. Syst. Biol. 52: 206–228. 12746147 10.1080/10635150390192726

[bib16] Haynes G. D. Latch E. K., 2012 Identification of novel single nucleotide polymorphisms (SNPs) in deer (Odocoileus spp.) using the BovineSNP50 BeadChip. PLoS One 7: e36536. 22590559 10.1371/journal.pone.0036536 PMC3348150

[bib17] He Y. Wang Z. H. Wang X. M., 2014 Genetic diversity and population structure of a Sichuan sika deer (Cervus sichuanicus) population in Tiebu Nature Reserve based on microsatellite variation. Dongwuxue Yanjiu 35: 528–536. 25465089 10.13918/j.issn.2095-8137.2014.6.528 PMC4790281

[bib18] Kerstens H. H. Crooijmans R. P. Veenendaal A. Dibbits B. W. Chin A. W. T. F., 2009 Large scale single nucleotide polymorphism discovery in unsequenced genomes using second generation high throughput sequencing technology: applied to turkey. BMC Genomics 10: 479. 19835600 10.1186/1471-2164-10-479 PMC2772860

[bib19] Kraus R. H. Kerstens H. H. Van Hooft P. Crooijmans R. P. Van Der Poel J. J., 2011 Genome wide SNP discovery, analysis and evaluation in mallard (Anas platyrhynchos). BMC Genomics 12: 150. 21410945 10.1186/1471-2164-12-150 PMC3065436

[bib20] Krojerova-Prokesova J. Barancekova M. Voloshina I. Myslenkov A. Lamka J., 2013 Dybowski’s sika deer (Cervus nippon hortulorum): genetic divergence between natural primorian and introduced Czech populations. J. Hered. 104: 312–326. 23454911 10.1093/jhered/est006

[bib21] Langmead B. Salzberg S. L., 2012 Fast gapped-read alignment with Bowtie 2. Nat. Methods 9: 357–359. 22388286 10.1038/nmeth.1923 PMC3322381

[bib22] Le S. Q. Durbin R., 2011 SNP detection and genotyping from low-coverage sequencing data on multiple diploid samples. Genome Res. 21: 952–960. 20980557 10.1101/gr.113084.110 PMC3106328

[bib23] Li H., 2011 A statistical framework for SNP calling, mutation discovery, association mapping and population genetical parameter estimation from sequencing data. Bioinformatics 27: 2987–2993. 21903627 10.1093/bioinformatics/btr509 PMC3198575

[bib24] Lu X. P. Wei F. W. Li M. Yang G. Liu H., 2006 Genetic diversity among Chinese sika deer (Cervus nippon) populations and relationships between Chinese and Japanese sika deer. Chin. Sci. Bull. 51: 433–440.

[bib25] Malhis N. Jones S. J. M., 2010 High quality SNP calling using Illumina data at shallow coverage. Bioinformatics 26: 1029–1035. 20190250 10.1093/bioinformatics/btq092

[bib26] McCullough E. D. R. Takatsuki S. Kaji K., 2009 Sika deer: biology and management of native and introduced populations, pp. 526–531 in *Sika Deer in Mainland China*, edited by McCullough D. R. Jiang Z.-G. Li C.-W. Springer, Tokyo.

[bib27] Patterson N. Price A. L. Reich D., 2006 Population structure and eigenanalysis. PLoS Genet. 2: 2074–2093. 10.1371/journal.pgen.0020190 PMC1713260 17194218

[bib28] Peterson B. K. Weber J. N. Kay E. H. Fisher H. S. Hoekstra H. E., 2012 Double digest RADseq: an inexpensive method for de novo SNP discovery and genotyping in model and non-model species. PLoS One 7: e37135. 22675423 10.1371/journal.pone.0037135 PMC3365034

[bib29] Powell J. H. Amish S. J. Haynes G. D. Luikart G. Latch E. K., 2016 Candidate adaptive genes associated with lineage divergence: identifying SNPs via next-generation targeted resequencing in mule deer (Odocoileus hemionus). Mol. Ecol. Resour. 16: 1165–1172. 27438092 10.1111/1755-0998.12572

[bib30] Pritchard J. K. Stephens M. Donnelly P., 2000 Inference of population structure using multilocus genotype data. Genetics 155: 945–959. 10835412 10.1093/genetics/155.2.945 PMC1461096

[bib31] Seabury C. M. Bhattarai E. K. Taylor J. F. Viswanathan G. G. Cooper S. M., 2011 Genome-wide polymorphism and comparative analyses in the white-tailed deer (Odocoileus virginianus): a model for conservation genomics. PLoS One 6: e15811. 21283515 10.1371/journal.pone.0015811 PMC3023705

[bib32] Sheng H. L. Ohtaishi N., 1993 *Deer of China: Biology and Management*. Elsevier Science, Amsterdam.

[bib33] Szyda J. Fraszczak M. Mielczarek M. Giannico R. Minozzi G., 2015 The assessment of inter-individual variation of whole-genome DNA sequence in 32 cows. Mamm. Genome 26: 658–665. 26475143 10.1007/s00335-015-9606-7 PMC4653241

[bib34] Takiguchi H. Tanaka K. Ono K. Hoshi A. Minami M., 2012 Genetic variation and population structure of the Japanese sika deer (Cervus nippon) in the Tohoku District based on mitochondrial D-loop sequences. Zoolog. Sci. 29: 433–436. 22775251 10.2108/zsj.29.433

[bib35] Tamate H. B. Okada A. Minami M. Ohnishi N. Higuchi H., 2000 Genetic variations revealed by microsatellite markers ina small population of the sika deer (Cervus nippon)on Kinkazan Island, northern Japan. Zoolog. Sci. 17: 47–53. 18494571 10.2108/zsj.17.47

[bib36] Thevenon S. Thuy L. T. Ly L. V. Maudet F. Bonnet A., 2004 Microsatellite analysis of genetic diversity of the Vietnamese sika deer (Cervus nippon pseudaxis). J. Hered. 95: 11–18. 14757725 10.1093/jhered/esh001

[bib37] Wang W. Yan H. Yu J. Yi J. Qu Y., 2017 Discovery of genome-wideSNPs by RAD-seqand the genetic diversity of captive hog deer (Axis porcinus). PLoS One 12: e0174299. 28323863 10.1371/journal.pone.0174299 PMC5360274

[bib38] Wu H. Wan Q. H. Fang S. G., 2004 Two genetically distinct units of the Chinese sika deer (Cervus nippon): analyses of mitochondrial DNA variation. Biol. Conserv. 119: 183–190.

[bib39] Wu H. Wan Q. H. Fang S. G. Zhang S. Y., 2005 Application of mitochondrial DNA sequence analysis in the forensic identification of Chinese sika deer subspecies. Forensic Sci. Int. 148: 101–105. 15639603 10.1016/j.forsciint.2004.04.072

[bib40] Wu P. J. Zhang E. D., 2001 The resource conservation and utilization of wild sika deer in China. Zhong Yao Cai 24: 552–554 (in Chinese). 11715188

[bib42] Zhu L. Zhang X. Deng C. Ding J. Ren Y., 2016 Comparative genomics and metagenomics analyses of endangered Père David’s deer (Elaphurus davidianus) provide insights into population recovery. bioRxiv DOI: 10.1101/073528.

